# miR‐127‐3p Inhibits Cell Stemness and Docetaxel Resistance in Triple‐Negative Breast Cancer by Targeting KIF3B

**DOI:** 10.1002/kjm2.70113

**Published:** 2025-10-29

**Authors:** Zi‐Chao He, Jun‐Jie Zhao, Ting Yan

**Affiliations:** ^1^ Department of General Surgery The Fourth Hospital of Changsha (Integrated Traditional Chinese and Western Medicine Hospital of Changsha, Changsha Hospital of Hunan Normal University) Changsha Hunan China

**Keywords:** docetaxel, drug resistance, KIF3B, miR‐127‐3p, triple‐negative breast cancer

## Abstract

This study investigated the mechanism of miR‐127‐3p in tumor cell stemness and docetaxel (DTX) resistance in triple‐negative breast cancer (TNBC). hsa‐miR‐127‐3p and KIF3B levels were predicted using databases and validated in TNBC and paracancerous tissues. KM survival curves were plotted to analyze the effect of miR‐127‐3p on patient survival. Pearson's analysis was used to determine the correlation between miR‐127‐3p and KIF3B mRNA in cancer tissues. Drug‐resistant TNBC cell lines were established. After transfection, the cell viability, IC50, proliferation, migration, invasion, DTX resistance, apoptosis, and expression of the stemness markers SOX2, OCT4, and Nanog were detected. Databases were used to predict the downstream targets of miR‐127‐3p. The starBase database and dual‐luciferase assay were used to predict and validate the binding relationship of miR‐127‐3p with KIF3B. Finally, the effect of miR‐127‐3p on transplanted tumors in TNBC nude mice was verified. miR‐127‐3p was expressed at low levels in TNBC tissues and was notably associated with shorter survival. Upregulation of miR‐127‐3p reduced TNBC cell stemness and DTX resistance. miR‐127‐3p targeted KIF3B. KIF3B overexpression averted the effect of miR‐127‐3p. miR‐127‐3p inhibited the growth of transplanted tumors in TNBC nude mice. Overall, miR‐127‐3p targets KIF3B, thereby reducing TNBC cell stemness and reversing DTX resistance.

## 1 Introduction

Breast cancer (BC) is the most prevalent malignancy and the primary cause of cancer‐associated death [1]. Triple‐negative BC (TNBC) is the most aggressive type of BC and is characterized by the absence of progesterone receptor, estrogen receptor, and human epidermal growth factor receptor 2 expression [2]. Compared with other subtypes, TNBC has a shorter survival time and a mortality rate of 40% within the first 5 years following diagnosis [3]. Current therapeutic options for TNBC are limited to surgery, chemotherapy, and radiotherapy, but some patients miss the opportunity for surgery at diagnosis [4]. Additionally, drug resistance often occurs several months after chemotherapy with docetaxel (DTX) and cisplatin [5]. Cancer stem cells can self‐renew and differentiate, thus generating heterogeneous cell populations in cancers [6]. Cancer cell stemness (a stem cell‐like phenotype in cancers) is linked to metastasis and chemoresistance [7]. Accordingly, analyzing the mechanism by which cancer cell stemness overcomes DTX resistance in TNBC is useful.

As reported, epigenetic factors, particularly microRNAs (miRs), are implicated in the occurrence and progression of BC [8]. Specifically, miR‐127‐3p exerts tumor‐repressing effects on many cancers, including ovarian, prostate, and gastric cancers [9, 10, 11]. Ectopic miR‐127‐3p sensitizes non‐small cell lung cancer cells to chemotherapy [12]. Importantly, miR‐127‐3p has also been reported to serve as a tumor repressor in TNBC [13]. Moreover, kinesin family member 3B (KIF3B) has been validated as a direct target gene of miR‐127‐3p in oral squamous cell carcinoma (OSCC) [14]. KIF3B participates in vesicle transport and membrane expansion during mitosis and orchestrates critical biological processes, such as cell migration and proliferation [15]. KIF3B has been reported to be highly expressed in various cancers, such as pancreatic cancer, colorectal cancer, and hepatocellular carcinoma [16, 17, 18]. Additionally, KIF3B is upregulated in BC tissues and cells, and its high expression is related to tumor recurrence and lymph node metastasis [19].

In this context, a hypothesis can be proposed that miR‐127‐3p may hinder the resistance of TNBC to DTX via KIF3B. Accordingly, this study was conducted to analyze whether miR‐127‐3p modulated cell stemness and then controlled DTX resistance in TNBC via KIF3B.

## 2 Materials and Methods

### 2.1 Bioinformatics Analysis

The starBase (https://rnasysu.com/encori/index.php) [20], dbDEMC (https://www.biosino.org/dbDEMC/index) [21], GEPIA2 (http://gepia2.cancer‐pku.cn/#index) [22], and GSCA (https://guolab.wchscu.cn/GSCA/#/) databases [23] were utilized to predict miR‐127‐3p and KIF3B expression in BC tissues. The starBase, TargetScan (https://www.targetscan.org/vert_80/) [24], miRDB (https://mirdb.org/mirdb/index.html) [25], and mirDIP (http://ophid.utoronto.ca/mirDIP/index.jsp#r) databases [26] were used to predict the downstream targets of miR‐127‐3p and plot Venn diagrams. The starBase database was used to predict the potential target binding sites of miR‐127‐3p and KIF3B.

### 2.2 Clinical Sample Collection and Survival Analysis

G*power 3.1.9.7 was used to estimate the sample size required for the paired samples *t*‐test, and the parameters were set as follows: effect size *dz* = 0.5, *α* err prob = 0.05, and power (1 − *β* err prob) = 0.95. The results showed that the total sample size required was at least 54 cases (Figure S1). Cancer tissue and normal adjacent tissue (NAT) samples were acquired from 78 patients with TNBC undergoing surgical treatment at the Fourth Hospital of Changsha (Integrated Traditional Chinese and Western Medicine Hospital of Changsha) from January 2018 to June 2022 for reverse transcription quantitative polymerase chain reaction (RT‐qPCR) and immunohistochemistry (IHC). The inclusion criteria for patients were as follows: (1) histopathological diagnosis of TNBC; (2) cancer tissue samples obtained from surgical resection of the primary tumor; and (3) complete follow‐up data. The exclusion criteria for patients were as follows: (1) combination with other malignant tumors; (2) metastatic TNBC or recurrence; and (3) failure to complete postoperative adjuvant chemotherapy according to the standard protocol. NAT samples were defined as normal breast tissue more than 5 cm away from cancerous tissue with no infiltration of cancer cells under microscopic observation. All patients were followed up regularly every 6 months after discharge for 3 years without any loss to follow‐up. Overall survival (OS) was calculated for all patients from the time of discharge to the time of death or the end of follow‐up. The study protocol followed the Declaration of Helsinki and was approved by the Ethics Committee of the Fourth Hospital of Changsha (Integrated Traditional Chinese and Western Medicine Hospital of Changsha) (approval number: CSSDSYY‐YXLL‐SC‐2023‐02‐19).

### 2.3 Cell Culture

The human TNBC cell lines MDA‐MB‐231 (SNL‐073) and MDA‐MB‐468 (SNL‐061) were cultured in a special medium for human BC cells (SNLM‐073) at 37°C with 100% air and saturated humidity. The cells and medium were purchased from SUNNCELL (Wuhan, Hubei, China). The 3rd passage cells at the logarithmic growth phase were detached with 0.25% trypsin (Procell Life, Wuhan, Hubei, China) and seeded in six‐well plates at 1 × 10^5^ cells/well. After reaching 70%–80% confluence, the cells were collected for further experiments.

### 2.4 DTX‐Resistant Cell Line Culture

Parental cells were treated with 1.0 μg/mL DTX for 48 h and then cultured in DTX‐free medium for 48 h. The process was repeated until the cells were stable in 1.0 μg/mL DTX medium. The entire process lasted for 3 months. The concentration of DTX was increased to 2.0, 4.0, 6.0, and 8.0 μg/mL. Finally, TNBC cells that survived stably after 8.0 μg/mL DTX treatment were used as DTX‐resistant cells (DTX/DR). Resistance index (RI) = IC50 of DTX/DR cell line/IC50 of parental cells.

### 2.5 3‐(4,5‐Dimethyl‐2‐Thiazolyl)‐2,5‐Diphenyl‐2‐H‐Tetrazolium Bromide (MTT) Assay

The MTT assay was utilized for the assessment of cell viability. Parental cells were exposed to different doses of DTX (1.0, 2.0, 4.0, 6.0, and 8.0 μg/mL), and primary drug‐resistant cells (P0) and 10th (P10)‐ and 20th (P20)‐passage drug‐resistant cells were exposed to different doses of DTX (5.0, 10.0, 20.0, 30.0, and 40.0 μg/mL). IC50 values were calculated separately. DTX/DR cells were treated with DTX at IC50 values in subsequent experiments.

### 2.6 Cell Transfection and Grouping

The miR‐127‐3p mimic (mimic miR), mimic NC, miR‐127‐3p inhibitor (inhi miR), inhibitor NC (inhi NC), pcDNA3.1‐KIF3B (oe‐KIF3B), and pcDNA3.1‐NC (oe‐NC) (all from GenePharma, Shanghai, China) were transfected into DTX/DR cells at 3.75 μL/mL, and subsequent experiments were carried out 24 h after transfection.

TNBC cells were categorized into WT (parental cells), DTX/DR (drug‐resistant cells), DTX/DR + mimic NC and DTX/DR + mimic miR (drug‐resistant cells transfected with miR‐127‐3p mimic or mimic NC), DTX/DR + inhi NC and DTX/DR + inhi miR (drug‐resistant cells transfected with miR‐127‐3p inhibitor or inhibitor NC), DTX/DR + mimic miR + oe‐NC and DTX/DR + mimic miR + oe‐KIF3B (drug‐resistant cells cotransfected with miR‐127‐3p mimic and either oe‐NC or oe‐KIF3B).

### 2.7 RT‐qPCR

Tissue and total cellular RNA was extracted using TRIzol reagent (Invitrogen Inc., Carlsbad, CA, USA) and then transcribed into cDNA using a PrimeScript RT Kit (Takara, China) and TaqMan MicroRNA RT Kit (Thermo Fisher Scientific Inc., China). Then, qPCR was performed on an ABI 7900HT Rapid PCR Real‐Time System (Applied Biosystems, Foster City, CA, USA) using SYBR Premix Ex Taq II (Takara) under pre‐denaturation at 95°C for 10 min, denaturation at 95°C for 10 s, annealing at 60°C for 20 s, and extension at 72°C for 34 s for 40 cycles. U6 was the internal reference for miR, and β‐actin was the internal reference for KIF3B. The data were computed via the $2^{-\Delta \Delta C_{t}}$ method. The primer sequences are listed in Table 1.

**TABLE 1:** Primer sequences for RT‐qPCR.

Genes	Sequences
hsa‐miR‐127‐3p	F: 5′‐ACACTCCAGCTGGGTCGGATCCGTCTGAGC‐3′
	R: 5′‐TGGTGTCGTGGAGTCG‐3′
mmu‐miR‐127‐3p	F: 5′‐CTTATCGGATCCGTCTGAGC‐3′
	R: 5′‐CAGTGCAGGGTCCGAGGTAT‐3′
U6	F: 5′‐GCTTCGGCAGCACATATACTAA‐3′
	R: 5′‐AACGCTTCACGAATTTGCGT‐3′
KIF3B	F: 5′‐ATTCAGCAGCAGATGGAGAGT‐3′
	R: 5′‐CTTGTATCCTACGGCAGAGACT‐3′
β‐Actin	F: 5′‐AAATCGTGCGTGACATTAA‐3′
	R: 5′‐CTCGTCATACTCCTGCTTG‐3′

*Note*: F, forward; R, reverse.

### 2.8 Cell Counting Kit‐8 (CCK‐8) Assay

The cells were grown in 96‐well plates at a density of 1 × 10^5^ cells/well, and the plates were precultured at 37°C with 100% air. CCK‐8 reagent (C0039, Beyotime, Shanghai, China) was used to detect cell proliferation. Then, 10 μL of CCK‐8 solution was added to each well for a 1‐h incubation. The absorbance at 450 nm was measured at 0, 24, 48, and 72 h of incubation using an ELx800 Reader (Bio‐Tek Instruments Inc., Winooski, VT, USA).

### 2.9 Colony Formation Assay

The cells were grown at 5 × 10^2^ cells/well in six‐well plates for 14 days. After the cells were fixed with 4% paraformaldehyde (P0099, Beyotime) and dyed with 1% crystal violet (C0121, Beyotime), the plaques were viewed under a bright field microscope (Olympus, Japan) and quantitatively analyzed using ImageJ (NIH, Bethesda, ML, USA).

### 2.10 Flow Cytometry

Apoptosis was examined by Annexin V‐fluorescein isothiocyanate/propidium iodide (FITC/PI) double staining. Briefly, 0.25% trypsin digestion and centrifugation were performed to harvest the cells. Following three washes with PBS, the cells were resuspended in binding buffer. The cells were cultured with Annexin V‐FITC and PI for 15–20 min in the dark using Annexin V‐FITC/PI kits (Elabscience, Houston, TX, USA) and detected within 1 h by flow cytometry (Aceabio, San Diego, CA, USA) for apoptosis.

### 2.11 Transwell Assay

Cell invasion was tested using Transwell chambers prefilled with Matrigel (BD Biosciences, San Diego, CA, USA), whereas the Transwell chambers used for cell migration assessment did not require Matrigel. The cells were starved in serum‐free medium for 24 h and then resuspended in medium containing 1% FBS. The upper chamber contained 1 × 10^5^ cells, and then 600 μL of complete medium supplemented with 10% FBS was added to the lower chamber for 24 h. Next, the uncrossed cells in the upper chamber were removed with a cotton swab, fixed with 4% paraformaldehyde, and dyed with 0.1% crystal violet. Five random fields of view were observed, and the cells were counted under an inverted microscope (Olympus, Japan) to obtain the average value.

### 2.12 Mammosphere Assay

A mammosphere assay [27] was performed. Briefly, 100 cells/well were cultured in single‐cell suspensions in 24‐well plates in serum‐free medium for 10 days. Breast microspheres were observed under a microscope (Olympus), and those ≥ 60 μm in size in each well were counted.

### 2.13 Western Blot (WB)

Total tissue and cellular proteins were extracted on ice using RIPA lysate (P0013E, Beyotime). The protein content of the supernatant was determined using BCA kits (P0011, Beyotime). Proteins (30 μg) were routinely isolated, transferred onto membranes, sealed, and then incubated with primary antibodies against KIF3B (GTX35189, 1:1,000, GeneTex, Irvine, CA, USA, 85 kDa), SOX2 (GTX101507, 1:5,000, GeneTex, 34 kDa), OCT4 (GTX101497, 1:5,000, GeneTex, 39 kDa), and Nanog (GTX100863, 1:1,000, GeneTex, 35 kDa) overnight at 4°C. After washing, the membranes were probed with the secondary antibody goat anti‐rabbit IgG (HRP) (GTX26721, 1:10,000, GeneTex) for 2 h. With β‐actin (GTX109639, 1:10,000, GeneTex, 42 kDa) as a reference, protein bands were examined using a chemiluminescence kit (ECL Plus, Life Technology) and analyzed in grayscale using ImageJ software.

### 2.14 Dual‐Luciferase Assay

The complementary binding sequence of miR‐127‐3p to KIF3B and its mutant sequence was amplified, cloned, and inserted into pmiR‐GLO (E1330, Promega, CA, USA) to construct the WT plasmid (KIF3B‐WT) and the corresponding mutant plasmid (KIF3B‐MUT). Mimic NC or miR‐127‐3p mimic was cotransfected into TNBC cells with the KIF3B‐WT or KIF3B‐MUT plasmid (GenePharma) using Lipofectamine 3000. After 48 h, luciferase activity was examined.

### 2.15 RNA Immunoprecipitation (RIP) Assay

A RIP assay was performed with anti‐Ago1 (Abcam, Cambridge, MA, USA) or IgG (Sigma‐Aldrich) [28]. Briefly, 24 h after transfection with miR‐127‐3p mimic or mimic NC, the cells were lysed in complete RIP lysate. Next, 100 μL of cell lysate was mixed with magnetic beads containing human anti‐Ago1 or IgG for 2 h at 4°C. Protein was digested with proteinase K, bound RNA was isolated with TRIzol (Invitrogen), and KIF3B expression was analyzed via RT‐qPCR.

### 2.16 Laboratory Animals

Female BALB/c nude mice (4 weeks old) from SLAC (Shanghai, China) were acclimatized for 1 week prior to the experiments and were maintained at 24°C–26°C with 50%–60% humidity and free access to food and water. The animal experiments were approved by the Animal Ethics Committee of the Fourth Hospital of Changsha (Integrated Traditional Chinese and Western Medicine Hospital of Changsha).

### 2.17 Tumor Xenograft Models

BALB/c nude mice were randomized into four groups (*n* = 6/group): WT + mimic NC, WT + mimic miR, DTX/DR + mimic NC, and DTX/DR + mimic miR. Tumor xenograft models were established as described previously [29]. Briefly, MDA‐MB‐231 WT and DTX/DR cells stably transfected with miR‐127‐3p mimic or mimic NC (5 × 10^6^) were injected subcutaneously into the right axilla of nude mice, and the growth of the nude mice and subcutaneously implanted tumors was closely monitored. After 10 days, 25 mg/kg DTX or an equivalent amount of PBS was injected intraperitoneally once a week. The width and length of the tumors were recorded every 7 days, and the tumor volume ((length × width^2^)/2) was computed. After 28 days of tumor formation, the mice were euthanized by an intraperitoneal overdose of 3% pentobarbital sodium (100 mg/kg), and the xenograft tumors were harvested, weighed, and placed in liquid nitrogen for freezing and storage for subsequent experiments.

### 2.18 IHC

Tissues were routinely fixed, dehydrated, embedded, and sliced into 5‐μm‐thick sections. Following routine dewaxing, rehydration, antigen retrieval, and inactivation of endogenous enzymes, the sections were probed with primary antibody against Ki67 (GTX16667, 1:100, GeneTex) at 4°C overnight. After the samples were washed, they were incubated with a secondary goat anti‐rabbit IgG antibody (HRP) (GTX26721, 1:1,n000, GeneTex) for 30 min. Nuclear staining with DAB (Sigma) was performed, and the sections were counterstained with hematoxylin. IHC was performed with a kit (Golden Bridge, Beijing, China). Positive sites were brownish yellow.

### 2.19 Statistical Analysis

All the data were analyzed and graphed with GraphPad Prism 9.5.0. The Shapiro–Wilk test was used to check for a normal distribution, and all the data that conformed to a normal distribution were presented as mean ± standard deviation. Pairwise comparisons were made via the *t* test for independent or paired samples, and multigroup comparisons were made via one‐way ANOVA and Tukey's multiple comparisons test. Pearson analysis of the link between miR‐127‐3p and KIF3B was conducted in the cancer tissues of patients with TNBC. Kaplan–Meier (KM) curves and the log‐rank test were used to analyze the effect of miR‐127‐3p expression on the survival prognosis of patients with TNBC. *p* < 0.05 from two‐sided tests denoted statistical significance.

## 3 Results

### 3.1 miR‐127‐3p is expressed at low levels in TNBC tissues and is associated with shorter survival

**FIGURE 1:**
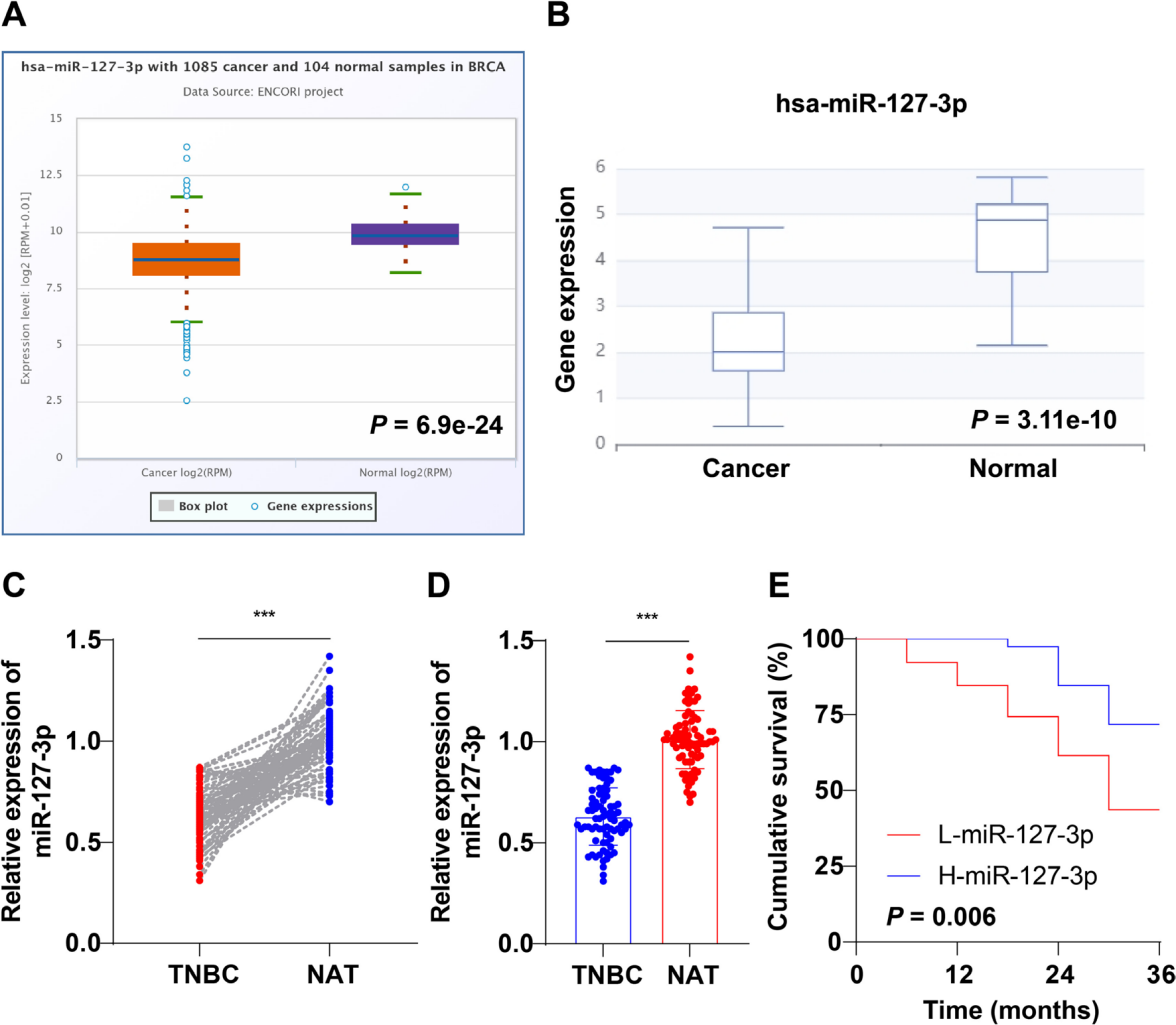
miR‐127‐3p is expressed at low levels in TNBC tissues and is associated with shorter survival. (A, B) The starBase and dbDEMC databases were used to predict hsa‐miR‐127‐3p expression in BC tissues; (C, D) Cancer tissues and NATs from 78 patients with TNBC were collected, and miR‐127‐3p expression was detected by RT‐qPCR; (E) The effect of miR‐127‐3p on the survival of patients with TNBC was analyzed by KM survival curves. Independent samples *t* tests were used in (A, B), paired samples *t* tests were used in (C‐D), and log‐rank tests were used in (E). ***p < 0.001.

First, the starBase and dbDEMC databases revealed that hsa‐miR‐127‐3p was strongly underexpressed in BC tissues (Figure 1A,B, all *p* < 0.001). Subsequently, RT‐qPCR revealed that the level of miR‐127‐3p was markedly lower in TNBC tissues than in NATs (Figure 1C,D, *p* < 0.001). Based on the median miR‐127‐3p (0.62), all patients were allocated into the H‐miR‐127‐3p group (> 0.62, *n* = 39) and the L‐miR‐127‐3p group (< 0.62, *n* = 39). KM survival curves revealed that the OS of patients with TNBC in the L‐miR‐127‐3p group was notably shorter than that in the H‐miR‐127‐3p group (Figure 1E, *p* < 0.01). Briefly, miR‐127‐3p was expressed at low levels in TNBC tissues and was notably associated with shorter survival.

### 3.2 miR‐127‐3p Upregulation Inhibits TNBC Cell Stemness and Reverses DTX Resistance

**FIGURE 2:**
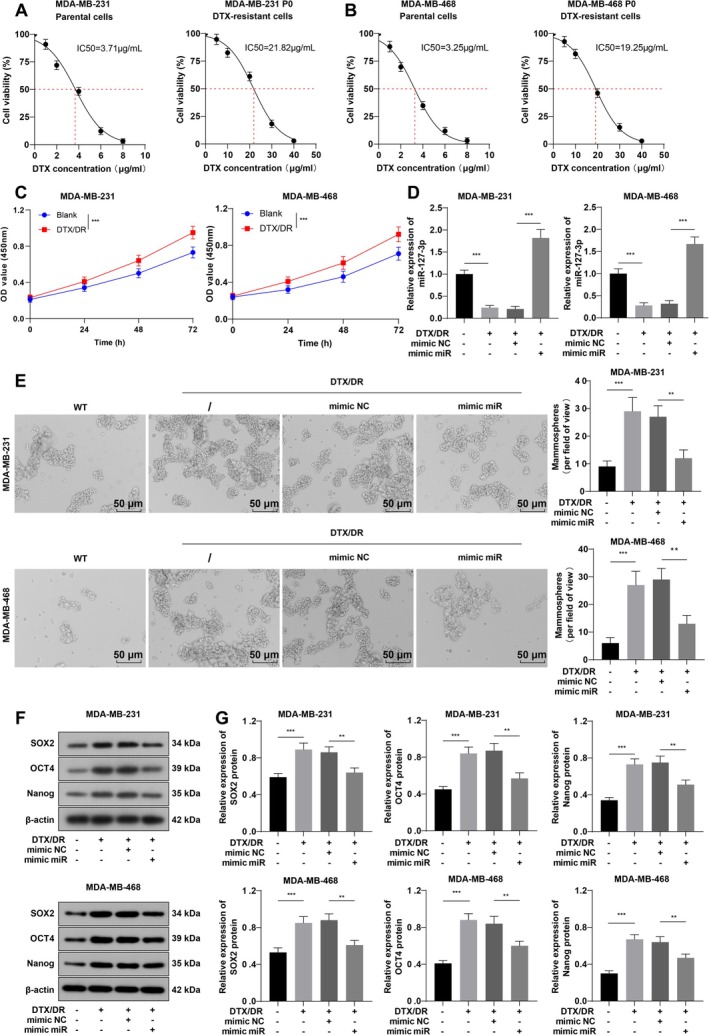
Upregulation of miR‐127‐3p inhibits TNBC cell stemness. (A, B) MTT assay to determine the IC50 values of parental cells and primary drug‐resistant cells (P0); (C) CCK‐8 assay for cell proliferation; (D) RT‐qPCR assay for miR‐127‐3p expression; (E) Mammosphere assay for sphere‐forming ability; (F, G) WB assay for the expression levels of the cell stemness markers SOX2, OCT4, and Nanog. The cellular experiments were repeated three times, the data were presented as mean ± standard deviation, and multigroup comparisons were analyzed by one‐way ANOVA with Tukey's test. **p < 0.01 and ***p < 0.001.

MDA‐MB‐231 and MDA‐MB‐468 cells were treated with DTX to establish TNBC drug‐resistant cell lines (DTX/DR). According to the calculation with GraphPad Prism 9.5.0 software, the IC50 values of DTX in parental and drug‐resistant MDA‐MB‐231 cells were 3.71 and 21.82 μg/mL, respectively, whereas the IC50 values of DTX in parental and drug‐resistant MDA‐MB‐468 cells were 3.25 and 19.25 μg/mL, respectively (Figure 2A,B). The RIs were 5.88 and 5.92 based on the IC50 values, respectively. The results of the CCK‐8 assay revealed that the proliferation of DTX/DR cells was greatly enhanced (Figure 2C, *p* < 0.001). RT‐qPCR revealed that the level of miR‐127‐3p in DTX/DR cells was significantly reduced (Figure 2D, *p* < 0.001). Additionally, to further validate the stability of drug‐resistant TNBC cells, the constructed drug‐resistant TNBC cells were passaged 10 and 20 times, after which the IC50 values of the P10/P20 drug‐resistant cells were measured. The results demonstrated that the IC50 values of P10 and P20 drug‐resistant MDA‐MB‐231 cells were 20.19 and 19.48 μg/mL, respectively. The IC50 values of the P10 and P20 drug‐resistant MDA‐MB‐468 cells were 18.17 and 17.10 μg/mL, respectively, which were all greater than 85% of the IC50 value of the P0 drug‐resistant cells (Figure S2A,B). These results indicated that the drug resistance of the DTX/DR cell lines constructed in this study was long‐term stable.

**FIGURE 3:**
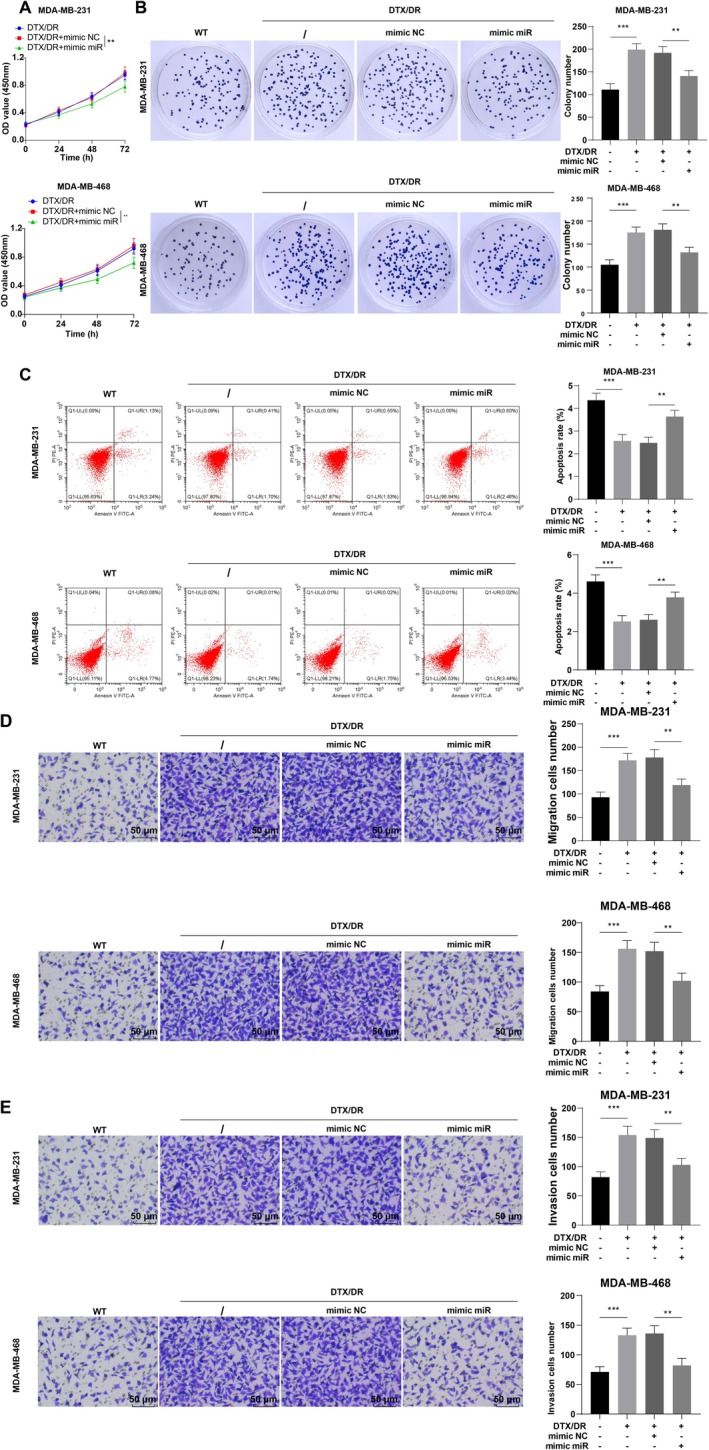
Upregulation of miR‐127‐3p promotes the sensitivity of TNBC cells to DTX. (A) CCK‐8 assay for cell proliferation; (B) Colony formation assay for cell sensitivity to DTX; (C) Flow cytometry for apoptosis; (D, E) Transwell assay for cell migration and invasion. The cellular experiments were repeated three times, the data were presented as mean ± standard deviation, and multigroup comparisons were analyzed by one‐way ANOVA with Tukey's test. **p < 0.01 and ***p < 0.001.

We subsequently transfected drug‐resistant cells with miR‐127‐3p mimic. RT‐qPCR was used to verify the transfection efficiency (Figure 2D, *p* < 0.001). The mammosphere assay revealed that the sphere‐forming ability of DTX/DR cells was greater than that of parental cells, whereas miR‐127‐3p overexpression significantly diminished the sphere‐forming ability of DTX/DR cells (Figure 2E, both *p* < 0.01). WB results revealed that SOX2, OCT4, and Nanog were elevated in DTX/DR cells compared with parental cells; miR‐127‐3p overexpression partially averted these changes (Figure 2F,G, all *p* < 0.01). Compared with those of parental cells, the proliferation, migration, and invasion of DTX/DR‐treated cells were notably greater, the number of colonies was greater, and the degree of apoptosis was lower, whereas miR‐127‐3p mimic transfection prevented these changes (Figure 3A–E, all *p* < 0.01). Overall, miR‐127‐3p upregulation repressed TNBC cell stemness and reversed DTX resistance.

### 3.3 miR‐127‐3p Targets KIF3B

**FIGURE 4:**
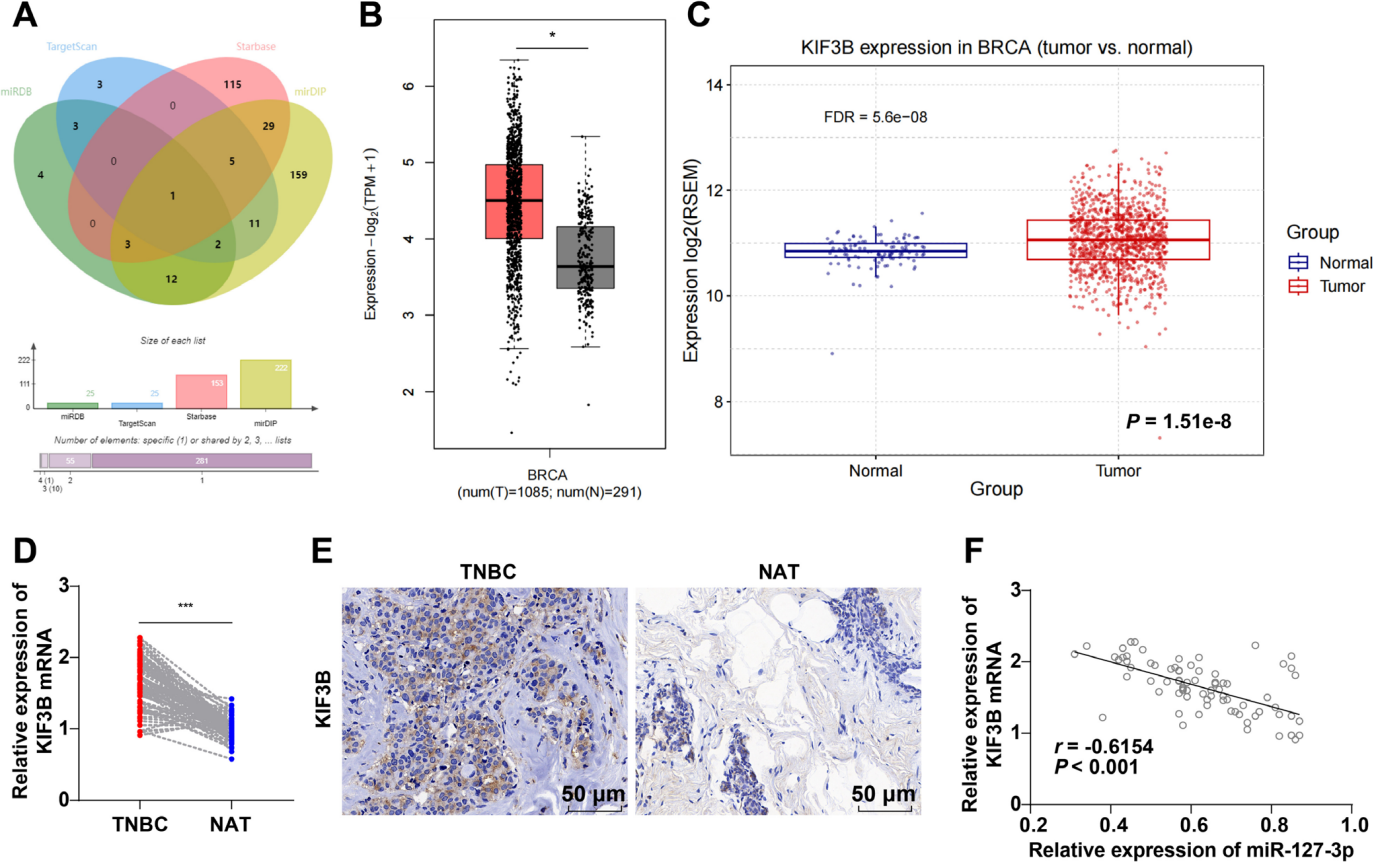
Bioinformatics and clinical analysis of the potential targeting relationship between miR‐127‐3p and KIF3B. (A) The starBase, TargetScan, miRDB and mirDIP databases were used to predict the downstream target genes of miR‐127‐3p, and Venn diagrams were generated. (B, C) The GEPIA2 and GSCA databases were used to predict KIF3B expression in BC tissues. (D, E) RT‐qPCR and IHC were used to detect KIF3B expression in the cancer tissues and NATs of patients with TNBC; (F) Pearson analysis of the correlation between miR‐127‐3p and KIF3B mRNA expression in the cancer tissues of patients with TNBC, where *r* is the correlation coefficient. Independent samples *t* tests were used in (B, C), and paired samples *t* tests were used in (D, E). *p < 0.05 and ***p < 0.001.

To investigate the regulatory mechanism by which miR‐127‐3p affects TNBC cell stemness and DTX resistance, the starBase, TargetScan, miRDB, and mirDIP databases were used to predict the downstream targets of miR‐127‐3p, plot Venn diagrams, and identify intersections. Only one candidate target, KIF3B, was obtained (Figure 4A). The GEPIA2 and GSCA databases predicted high KIF3B expression in BC tissues (Figure 4B,C, all *p* < 0.05). RT‐qPCR and IHC subsequently revealed that KIF3B was strongly upregulated in TNBC tissues compared with NATs (Figure 4D,E, *p* < 0.001). Finally, Pearson analysis revealed that miR‐127‐3p was significantly negatively linked to KIF3B mRNA expression in the cancer tissues of patients with TNBC (Figure 4F, *p* < 0.001).

**FIGURE 5:**
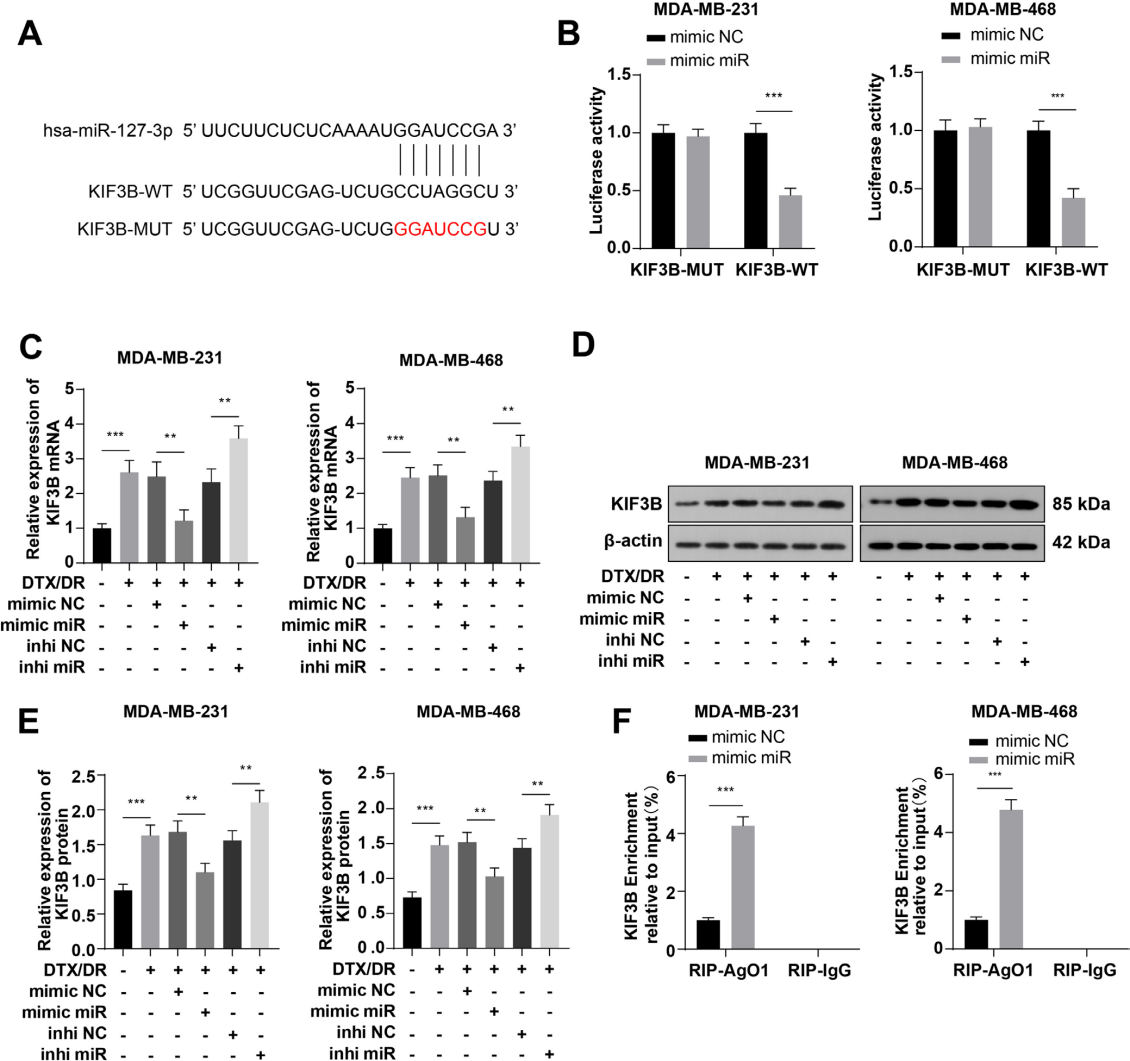
miR‐127‐3p targets KIF3B. (A) The starBase database was used to predict the potential binding site of miR‐127‐3p with KIF3B; (B) Dual‐luciferase reporter assay was used to validate the binding relationship between miR‐127‐3p and KIF3B; (C–E) RT‐qPCR and WB were used to detect KIF3B expression; (F) Quantitation of the recruited mRNAs of KIF3B to the miRNA complex immunoprecipitated with Ago1 by RIP analysis. The cell experiments were repeated three times, and the data were manifested as mean ± standard deviation and analyzed by one‐way ANOVA with Tukey's test. **p < 0.01 and ***p < 0.001.

Next, starBase database prediction identified a potential binding site for miR‐127‐3p with KIF3B (Figure 5A). A dual‐luciferase assay verified the binding relationship of miR‐127‐3p with KIF3B. Luciferase activity was notably reduced after cotransfection of the miR‐127‐3p mimic with KIF3B‐WT (Figure 5B, *p* < 0.001), whereas luciferase activity was not visibly altered after cotransfection of the miR‐127‐3p mimic and KIF3B‐MUT (*p* > 0.05). Subsequently, RT‐qPCR and WB revealed that KIF3B was upregulated in the DTX group, downregulated after miR‐127‐3p mimic transfection, and again upregulated in the DTX + inhi miR group (Figure 5C–E, all *p* < 0.01). Finally, the RIP assay revealed that miR‐127‐3p could directly interact with KIF3B (Figure 5F, *p* < 0.001). Overall, miR‐127‐3p targeted KIF3B.

### 3.4 miR‐127‐3p Targets KIF3B to Inhibit MDA‐MB‐231 Cell Stemness and Reverse DTX Resistance

**FIGURE 6:**
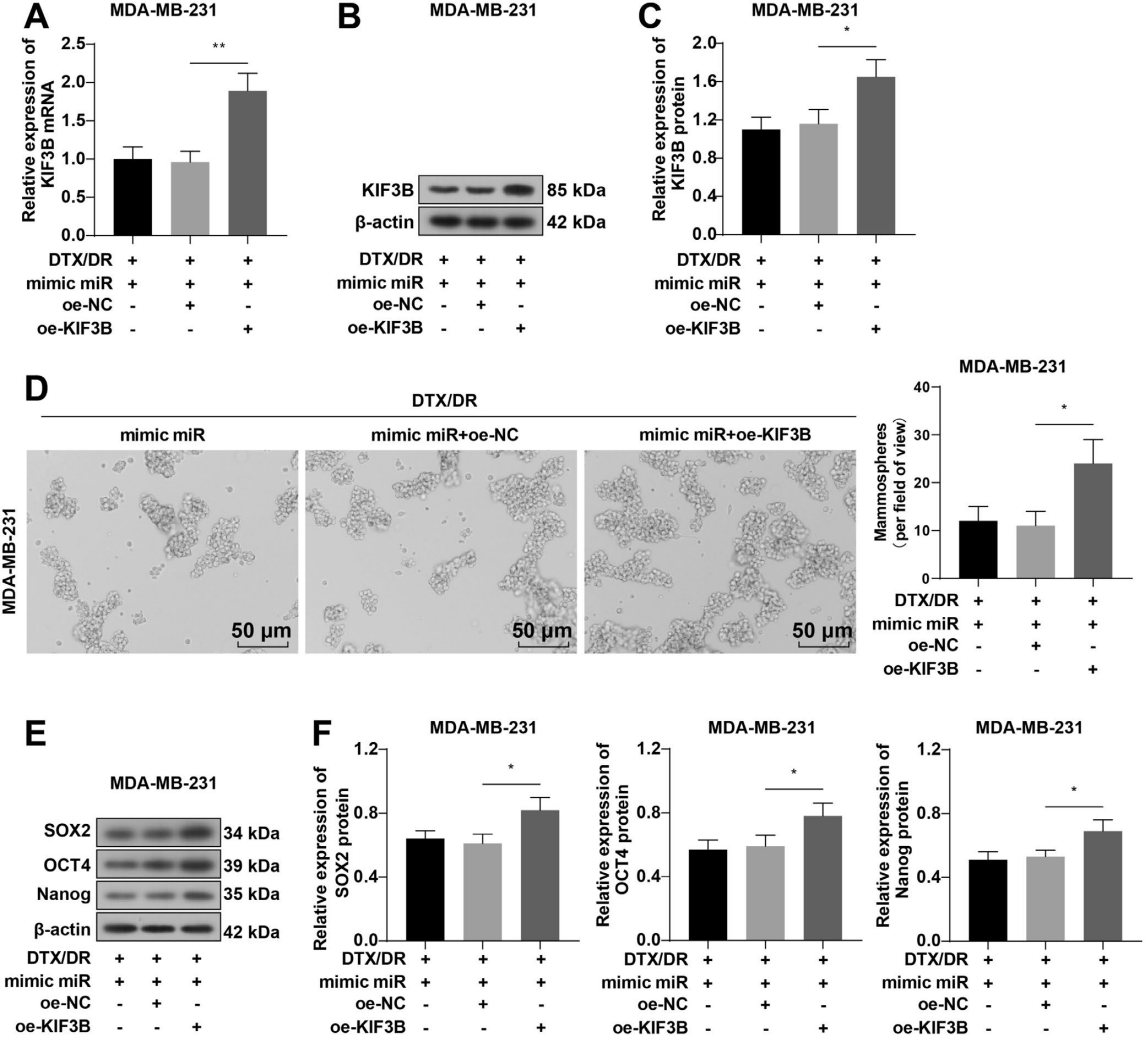
Overexpression of KIF3B partially reverses the inhibitory effect of miR‐127‐3p on MDA‐MB‐231 cell stemness. Drug‐resistant MDA‐MB‐231 cells were cotransfected with miR‐127‐3p mimic and oe‐KIF3B. (A–C) RT‐qPCR and WB for the detection of KIF3B overexpression efficiency; (D) Sphere formation assay; (E, F) WB for the detection of the protein levels of the cell stemness markers SOX2, OCT4 and Nanog. The cellular experiments were repeated three times, and the data were presented as mean ± standard deviation and analyzed by one‐way ANOVA with Tukey's test. *p < 0.05 and **p < 0.01.

**FIGURE 7:**
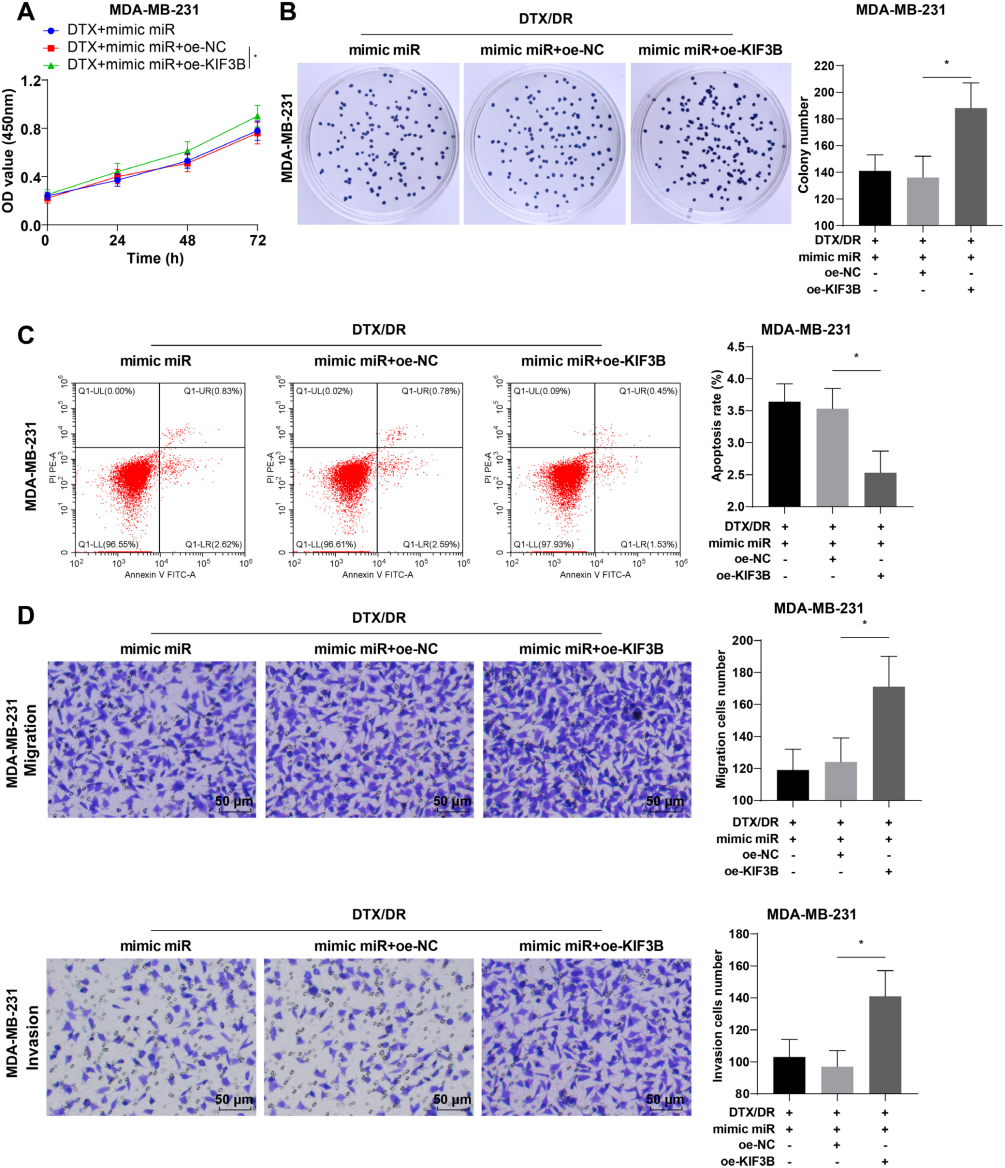
Overexpression of KIF3B partially reverses the inhibitory effect of miR‐127‐3p on DTX resistance in MDA‐MB‐231 cells. Drug‐resistant MDA‐MB‐231 cells were cotransfected with miR‐127‐3p mimic and oe‐KIF3B. (A) CCK‐8 assay for detecting cell proliferation; (B) Colony formation assay for assessing cell sensitivity to DTX; (C) Flow cytometry for testing apoptosis; (D) Transwell assay for determining cell migration and invasion. The cellular experiments were repeated three times, and the data were presented as mean ± standard deviation and analyzed by one‐way ANOVA with Tukey's test. *p < 0.05.

To verify that miR‐127‐3p affects TNBC cell stemness and DTX resistance by targeting KIF3B, we cotransfected drug‐resistant MDA‐MB‐231 cells with miR‐127‐3p mimic and oe‐KIF3B. RT‐qPCR and WB were used to verify the transfection efficiency. Compared with that in the DTX + mimic miR + oe‐NC group, KIF3B expression was greatly elevated in the DTX + mimic miR + oe‐KIF3B group (Figure 6A–C, all *p* < 0.05). The results of the sphere formation assay and WB revealed that the ability of the cells to form spheres was increased in the DTX + mimic miR + oe‐KIF3B group, and the expression of SOX2, OCT4, and Nanog was considerably increased (Figure 6D–F, all *p* < 0.05). After cotransfection of miR‐127‐3p mimic and oe‐KIF3B, cell growth was visibly enhanced, the number of colonies increased, and apoptosis was reduced (Figure 7A–D, all *p* < 0.05). Overall, miR‐127‐3p targeted KIF3B to repress TNBC cell stemness and reverse DTX resistance.

### 3.5 miR‐127‐3p Inhibits the Growth of Transplanted Tumors in TNBC Nude Mice

**FIGURE 8:**
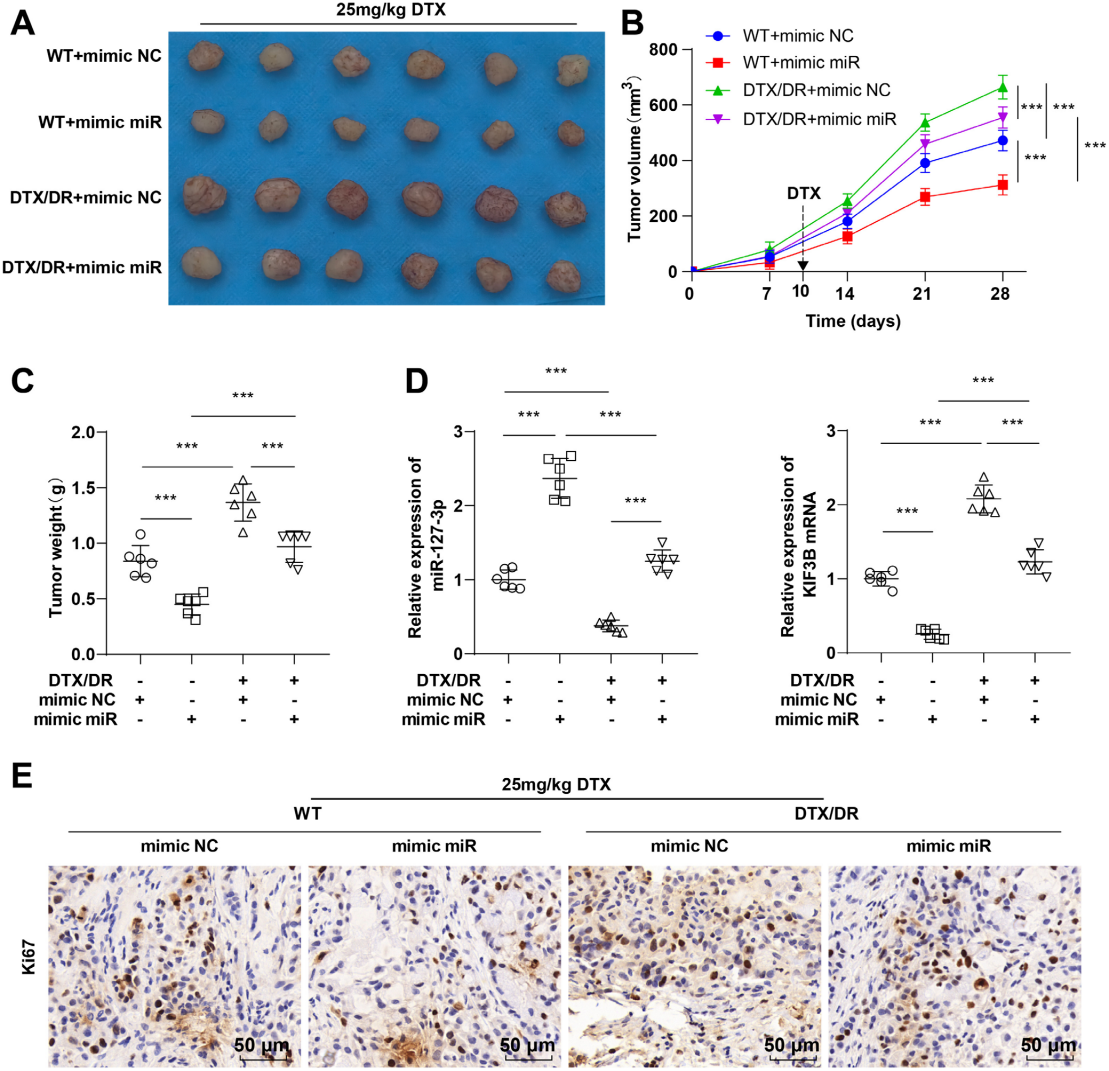
miR‐127‐3p inhibits the growth of transplanted tumors in TNBC nude mice. MDA‐MB‐231 WT and DTX/DR cells stably transfected with miR‐127‐3p mimic or mimic NC were injected subcutaneously into the right axilla of nude mice and given 25 mg/kg DTX intraperitoneally. (A) Tumor visualization; (B) Tumor volume; (C) Tumor weight; (D) RT‐qPCR for miR‐127‐3p and KIF3B mRNA expression; (E) IHC for Ki67 protein expression. *n* = 6, the data were presented as mean ± standard deviation and analyzed by one‐way ANOVA with Tukey's test. ***p < 0.001.

Finally, we explored the effect of miR‐127‐3p on TNBC in nude mice in vivo. KIF3B and Ki67 in the DTX/DR + mimic NC group were elevated, and tumor volume and weight were notably increased; miR‐127‐3p overexpression in both the MDA‐MB‐231 WT and DTX/DR groups significantly downregulated KIF3B and Ki67 and reduced tumor volume and weight. Compared with the WT + mimic miR group, the DTX/DR + mimic miR group presented notably greater expression of KIF3B and Ki67 and greater tumor volume and weight (Figure 8A–E, all *p* < 0.001). Overall, miR‐127‐3p inhibited the growth of transplanted tumors in TNBC nude mice.

## 4 Discussion

For TNBC, treatment options are limited, and chemotherapy remains the standard option for TNBC [30]. Unfortunately, with the progression of the disease, patients with TNBC frequently develop chemoresistance and metastasis [31]. Therefore, in recent years, enormous efforts have been devoted to ascertaining the molecular mechanisms underlying chemoresistance in TNBC with the purpose of identifying novel molecular targets. The present study revealed that miR‐127‐3p targeted KIF3B, thereby reducing TNBC cell stemness and reversing DTX resistance.

Notably, the anti‐oncogenic role of miR‐127‐3p has been demonstrated in many studies. A previous study revealed that a miR‐127‐3p mimic diminished the proliferation and invasion of glioblastoma cells [32]. Another study revealed that miR‐127‐3p expression was obviously poor in osteosarcoma and that ectopically expressed miR‐127‐3p impeded viability, antiapoptotic effects, migration, and invasion in osteosarcoma cells [33]. Research by Wan et al. revealed that miR‐127‐3p upregulation retarded the proliferation and migration of melanoma cells while accelerating their apoptosis [34]. Additionally, a prior study unraveled that miR‐127‐3p was expressed at low levels in BC tissues and that its upregulation depressed the proliferation and metastasis of BC cells [35]. Another study elucidated that miR‐127‐3p was clearly downregulated in BC tissues and was prominently linked to disease stage, indicating that miR‐127‐3p could serve as a potential biomarker for distinguishing BC and nontumor tissue samples [8]. Importantly, our data consistently revealed that miR‐127‐3p was poorly expressed in TNBC tissues and was strongly linked to shorter survival. Furthermore, Umeh‐Garcia et al. reported that a miR‐127 prodrug reduced the viability, motility, and chemoresistance of TNBC cells and restricted the TNBC stem cell population [36], underscoring the repressive impacts of miR‐127 on the progression and chemoresistance of TNBC. Similarly, our data revealed that miR‐127‐3p expression was low in DTX‐resistant DTX/DR cells and that miR‐127‐3p mimic weakened sphere‐forming, proliferating, migrating, and invasive capabilities and declined the expression of cell stemness markers (SOX2, OCT4, and Nanog) in DTX/DR cells. Moreover, miR‐127‐3p mimic constrained the growth of transplanted tumors in TNBC nude mice. Hence, miR‐127‐3p overexpression hindered cell stemness and subsequently reversed DTX resistance in TNBC cells.

Given that miR‐127‐3p directly targets KIF3B in OSCC [14], the present study further investigated whether KIF3B was involved in the inhibitory effects of miR‐127‐3p on cell stemness and DTX resistance in TNBC. In our study, database prediction demonstrated that KIF3B was targeted by KIF3B, which was validated through dual‐luciferase and RIP assays. KIF3B functions as an oncogene in an array of cancers. For instance, KIF3B expression is elevated in colon cancer tissues, and decreased KIF3B expression represses the proliferation, anti‐apoptosis, and invasion of colon cancer cells [37]. KIF3B downregulation inhibits osteosarcoma cell invasion and migration [38]. A prior study reported high KIF3B expression in BC tissues [39], corroborating that KIF3B is upregulated in TNBC tissues, as observed in our study. Another study revealed that KIF3B loss protected against the EMT of BC cells [19]. Nevertheless, only one study has assessed the influence of KIF3B on therapeutic resistance in cancers, which unraveled that KIF3B downregulation increased the sensitivity of ESCC cells to radiotherapy [40]. The present study further revealed that KIF3B overexpression potentiated DTX resistance in TNBC, as evidenced by increased proliferation, invasion, migration, and anti‐apoptosis in DTX/DR TNBC cells after oe‐KIF3B treatment in the existence of miR‐127‐3p mimic. Innovatively, our study also delved into the impact of KIF3B on cell stemness in TNBC to further evaluate the role of KIF3B in chemoresistance. The results demonstrated that oe‐KIF3B treatment nullified miR‐127‐3p mimic‐triggered reductions in sphere‐forming capabilities and cell stemness markers in DTX/DR cells. Overall, these findings unveiled that overexpressing KIF3B potentiated the resistance of TNBC cells to DTX.

In conclusion, miR‐127‐3p was diminished in patients with TNBC and DTX‐resistant TNBC cells, and miR‐127‐3p overexpression protected against TNBC cell stemness via the targeting of KIF3B, which resulted in the suppression of DTX resistance in TNBC. The current study provides new insights into the mechanisms underlying chemoresistance in TNBC and reveals the potential of miR‐127‐3p as a target for TNBC treatment. However, this study included a small clinical sample size and was a single‐center study, necessitating multicenter studies involving large sample sizes. Additionally, due to time, manpower, and funding constraints, we did not perform rescue experiments with miR‐127‐3p mimic and oe‐KIF3B in xenograft tumors, which calls for further studies.

## Ethics Statement

The study protocol followed the Declaration of Helsinki and was ratified by the Ethics Committee of The Fourth Hospital of Changsha (Integrated Traditional Chinese and Western Medicine Hospital of Changsha). All patients were fully aware of the study objective, with signature on the informed consent forms obtained from them (Ethics approval number: CSSDSYY‐YXLL‐SC‐2023‐02‐19).

## Conflicts of Interest

The authors declare no conflicts of interest.

## Supporting information


**Figure S1:** Sample size estimation.

**Figure S2:** Validation of the stability of drug‐resistant TNBC cell lines. Drug‐resistant TNBC cell lines were constructed with the drug concentration escalation method and passaged 10 or 20 times. (A, B) MTT assay to examine the IC50 values of drug‐resistant cells at the 10th passage (P10) and 20th passage (P20). The cell experiments were repeated three times, and the data were expressed as mean ± standard deviation.

## Data Availability

The data that support the findings of this study are available from the corresponding author upon reasonable request.
