# Supplementary figures and images for: miR‐127‐3p Inhibits Cell Stemness and Docetaxel Resistance in Triple‐Negative Breast Cancer by Targeting KIF3B
Source: Kaohsiung J Med Sci. 2025 Oct 29;42(3):e70113. doi: 10.1002/kjm2.70113 (PMC12955890; doi:10.1002/kjm2.70113)

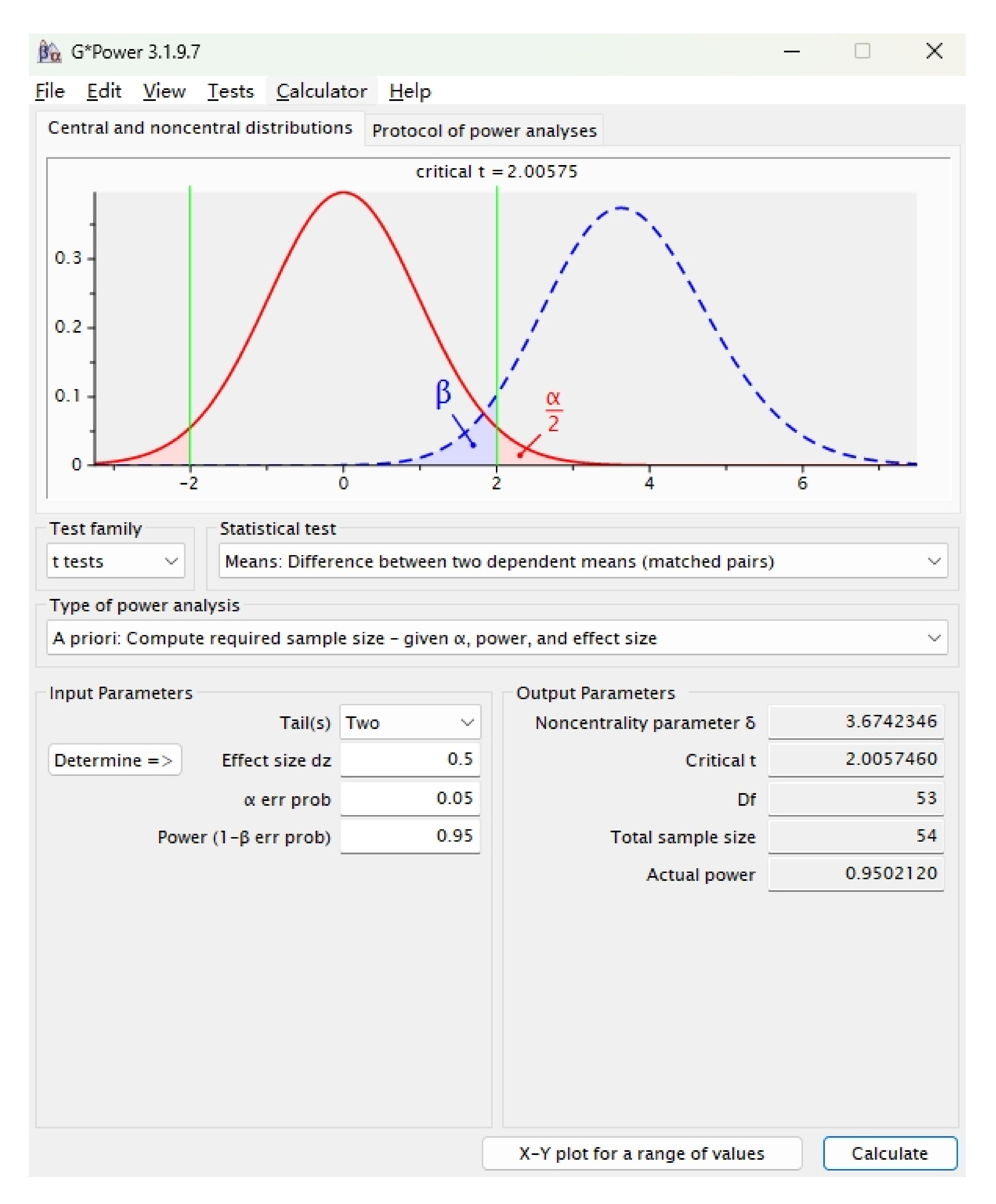

Supplement: Supplementary file 1 — Figure S1: Sample size estimation. [file KJM2-42-e70113-s002.tif]

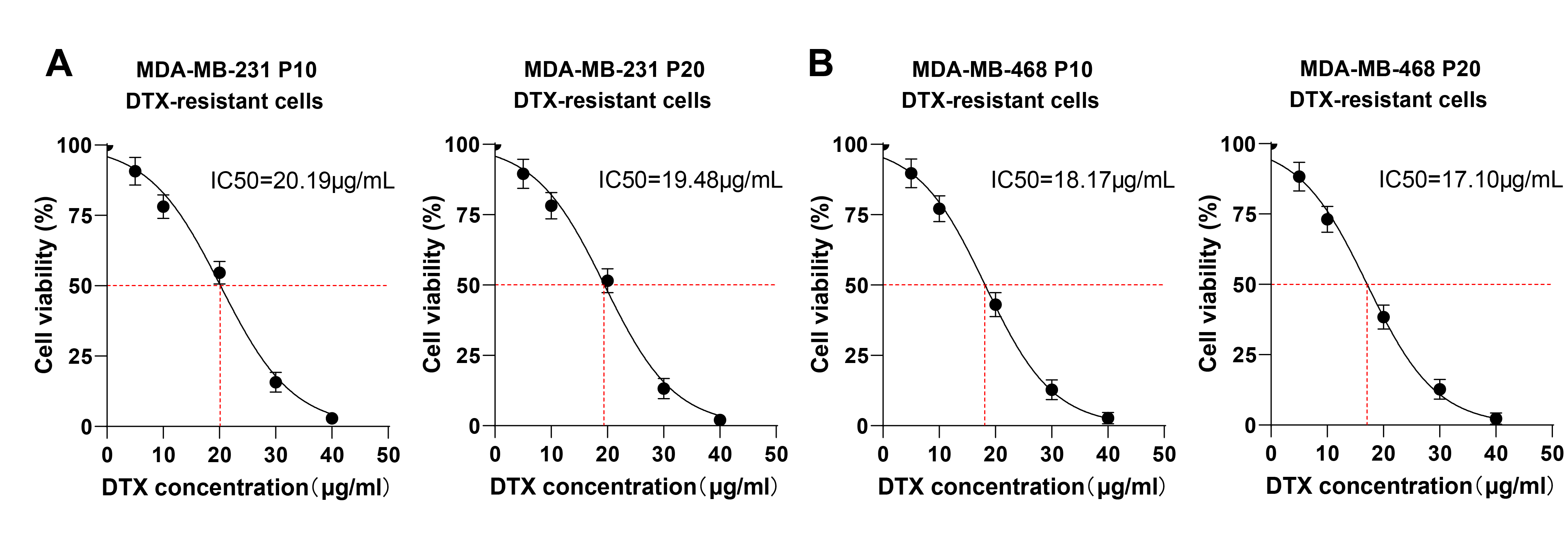

Supplement: Supplementary file 2 — Figure S2: Validation of the stability of drug‐resistant TNBC cell lines. Drug‐resistant TNBC cell lines were constructed with the drug concentration escalation method and passaged 10 or 20 times. (A, B) MTT assay to examine the IC50 values of drug‐resistant cells at the 10th passage (P10) and 20th passage (P20). The cell experiments were repeated three times, and the data were expressed as mean ± standard deviation. [file KJM2-42-e70113-s001.tif]
